# History Ever Repeats Itself

**Published:** 1900-05

**Authors:** 


### HISTORY EVER REPEATS ITSELF.

In many parts of our land the same general tenor of discussion is going on relative to bovine tuberculosis that has followed in the wake of every advanced movement in dealing with the various animal plagues that have devastated from time to time entire sections of our country, impoverished and bankrupted thousands of our live-stock growers and owners, placed embargoes on our food-products in every foreign market of the world, and halted industry and thrift, throttled progress and scientific investigation, and then charged Providence with their misfortune.

Live-stock journals, breeders' associations, and communities of live-stock owners are working themselves up to a high pitch, almost bursting with indignation, and arousing the opposition of thousands who, from ignorance and thoughtlessness of their own welfare, are easy victims to the delusive charges, the foundationless statements, the inflammatory headlines of journals, from whose editorial staffs better things were expected, as they tirade against their so-termed "Quarantine Craze against Tuberculosis." In their consuming zeal they are wholly forgetful of the record they are making in history repeating. As they will not learn from past blunders, when they cried out against the stamping-out methods of contagious pleuro-pneumonia, the quarantining against dourine, the rigid control of foot-and-mouth disease, the methods of control of Texas fever, hog cholera, glanders and other equally dangerous diseases. They would print their columns in blood-red ink against the removal of any of the methods extant to-day for the protection of their families against the dangers of Asiatic cholera, bubonic plague, yellow fever, smallpox, diphtheria, scarlet fever, and send up a hue and cry all over the land to halt typhoid fever and other diseases whose cause and methods of propagation are now fully determined; forgetful that all of these scourges of man and beast represent in the death-list as a total only a small percentage of the deaths all over the civilized world from tuberculosis in its many forms; oblivious of the facts that of the thousands of boasted cures, remedies, and nostrums *ad nauseam* that have come forth for its cure have added but little as a saving factor in the frightful percentage of 14 in every 100 deaths in the civilized world daily occurring from this disease. Newspaper clamor, the short-sighted ignorance of breeders' organizations, and indignation meetings stirred up by these thoughtless tirades will not turn back the wheels of progress, born of scientific investigation and the discovery of new truths; nor will they do more than temporarily halt the measures now so clearly outlined to control and in great measure eradicate this destructive disease. Self-preservation is the first law of man, and every State that shuts its doors to the admission of dangerous additions to its breeding and dairy herds starts right and will come out best in the end. Control measures of every kind will do more in this day of enlightenment for this disease in one year than has been accomplished in a score of years through medication. Quarantine measures in Western States and Territories would not be needful to-day had similar plans been in vogue the past twenty years during the enriching of their herds from Eastern and foreign sources, where tuberculosis prevails to a much greater extent.

